# Glutamatergic dysfunction, neuroplasticity, and redox status in the peripheral blood of patients with motor conversion disorders (functional movement disorders): a first step towards potential biomarkers discovery

**DOI:** 10.1038/s41398-023-02500-8

**Published:** 2023-06-17

**Authors:** Benedetta Demartini, Veronica Nisticò, Caroline Benayoun, Anna Chiara Cigognini, Roberta Ferrucci, Alessandra Vezzoli, Cinzia Dellanoce, Orsola Gambini, Alberto Priori, Simona Mrakic-Sposta

**Affiliations:** 1 Dipartimento di Scienze della Salute, Università degli Studi di Milano, Milano, Italy; 2 Unità di Psichiatria 52, Presidio San Paolo, ASST Santi Paolo e Carlo, Milano, Italy; 3 “Aldo Ravelli” Research Center for Neurotechnology and Experimental Brain Therapeutics, Università degli Studi di Milano, Milano, Italy; 4 Dipartimento di Psicologia, Università degli Studi di Milano – Bicocca, Milano, Italy; 5 III Clinica Neurologica, Presidio San Paolo, ASST Santi Paolo e Carlo, Milano, Italy; 6 Institute of Clinical Physiology - Milan, National Research Council (CNR), Milano, Italy

**Keywords:** Molecular neuroscience, Diagnostic markers

## Abstract

Functional movement disorders (FMD) are characterized by the presence of neurological symptoms that cannot be explained by typical neurological diseases or other medical conditions. First evidence showed that, compared to healthy controls (CTR), FMD patients presented increased levels of glutamate+glutamine in the anterior cingulate cortex/medial prefrontal cortex, and decreased levels of glutamate in the cerebrospinal fluid, suggesting that a glutamatergic dysfunction might play a role in FMD pathophysiology. In this study, 12 FMD patients and 20 CTR were recruited and underwent venous blood sampling and urine collection: levels of glutamate, BDNF, dopamine, oxidative stress, creatinine, neopterin, and uric acid were analyzed. Participants also underwent a psychometric assessment investigating depression, anxiety, and alexithymia. We found that levels of glutamate, BDNF, and dopamine were significantly lower in the blood of FMD patients than CTR. Glutamate and dopamine levels were positively associated with levels of alexithymia. Our findings give further evidence that glutamatergic dysfunction might be involved in the pathophysiology of FMD, possibly representing a biomarker of disease; moreover, since glutamatergic and dopaminergic systems are closely interconnected, our results might have a relevance in terms of treatment options for FMD patients.

## Introduction

Functional movement disorders (FMD) are part of the wide spectrum of functional neurological disorders (FND, also called conversion disorders), characterized by neurological symptoms of altered voluntary motor or sensory function that cannot be explained by typical neurological diseases or other medical conditions [1]. Despite their high prevalence and their impact on national health systems [2], their pathophysiology remains unclear, the diagnostic process is often long and challenging, and treatment options are not always effective. In the last decades, novel hypotheses based on the integration between psychology and neurobiology have been formulated, considering the neurobiological factors contributing to FND development and maintenance [3, 4]. Given that their diagnosis is not always straightforward, and that there are no reliable ways of determining prognosis or treatment response at first presentation, a great amount of work has been done in search of potential biomarkers of FMD. A recent review [5] showed that potentially reliable biomarkers can be found through electroencephalography and electromyography for functional myoclonus and tremor, two common manifestations of FMD; they also underline that several biomarkers often require access to time- and resource-consuming techniques, such as functional MRI (for a review see 4) and structural MRI [6]. Considerably fewer studies focused on the molecular level of analysis in FMD/FND. Recent evidence highlighted the presence of alterations in levels of glutamate, the major excitatory neurotransmitter, in patients with FMD [7, 8]: in a recent case-control study conducted with magnetic resonance spectroscopy (MRS), our group detected an increase of glutamate + glutamine (Glx) levels in the limbic system of patients suffering from FMD, with respect to healthy controls (CTR) [7]. In a subsequent study, we found significantly lower glutamate levels in the cerebrospinal fluid (CSF) of FMD patients, compared to CTR [8], again arguing in favor of a glutamatergic dysfunction in patients with FMD. Moreover, among the different neurotrophins involved in the development, survival, maintenance, and plasticity of neurons in the nervous system, Brain-Derived Neurotrophic Factor (BDNF) has been shown to be involved in the pathophysiology of different psychiatric illnesses [9], including FND: Deveci et al. [10] demonstrated that serum BDNF levels are significantly higher in patients diagnosed with conversion disorders than in CTR. Within other factors that may contribute to FND pathophysiology, the oxidative stress (OxS) is noteworthy, as the brain is greatly vulnerable to oxidative damage due to reactive oxygen species (ROS) unbalanced production. OxS occurs when free radicals, constantly produced in the tissues, reach too high concentrations. Under these conditions, O_2_ derivatives and peroxides can damage the various cell compartments. OxS is involved in pathogenic mechanisms of many psychiatric disorders, including depression, anxiety and schizophrenia [11], given the extreme vulnerability of the brain to oxidative damage. Nevertheless, markers of oxidative stress, such as ROS, 8-isoprostane (a metabolite of arachidonic acid) [12], thiols (representing in their oxidized forms the “redox status”) [13], nitrite (NO_3_) + nitrate (NO_2_) (NOx), inducible nitric oxide synthase (iNOS) and 3-nitrotyrosine (3-NT), have never been specifically assessed in FND.

Moreover, an important neurotransmitter involved in behavioral, cognitive, voluntary motor, motivational, attention, learning and mood circuits is dopamine. It is released centrally from the substantia nigra and exerts an inhibitory action on GABAergic neurons. It acts at the levels of the nuclei of the base, the corpus striatum, and the substantia nigra. Dopaminergic activity is finely regulated by many brain structures, such as the ventral subiculum of the hippocampus and the basolateral amygdala [14]. The role of dopamine is well-known not only in the pathogenesis of schizophrenia and Parkinson’s disease, but also in the pathogenesis of other psychiatric conditions, such as depression and anxiety. Nonetheless, it has never been assessed in the blood of patients with FND. Finally, creatinine, neopterin and uric acid have been recently associated with several neuropsychiatric conditions [15–17] but, again, they have never been studied in FND. Creatinine is an amino acid derivative, produced by the liver and used in the muscles to produce ATP for the purpose of muscle contraction. An increase in creatinine levels in the pre-frontal cortex has been shown to improve mood and well-being [15]. Neopterin is a catabolic product of guanosine triphosphate, a purine nucleotide. Recently it was argued that an increase in neopterin levels in biological fluids (e.g., serum, cerebrospinal fluid, or urine) strongly correlates with various neuropsychiatric disorders [16]. Uric acid is a molecule produced by the metabolism of purines: a study by Chen et al. associated the reduction in serum uric acid levels with a higher suicide rate in patients with major depressive disorder [17].

According to the evidence of these abnormalities in many neuropsychiatric disorders at level of brain network activity, connectivity, and specific anatomic areas of altered metabolic, and given the evidence of a potential role of glutamate and BDNF in the pathophysiology of FND, in this study we aimed to assess circulating levels of glutamate, BDNF, dopamine, oxidative stress biomarkers, creatinine, neopterin and uric acid in patients with FMD and in a control group of healthy subjects.

Deepening the knowledge of FMD, in terms of finding disease biomarkers and subsequently drawing specific treatments, might not only help defining potential mechanisms underlying the disorder, but also have clinical implications, both from a diagnostic and therapeutic angle: early diagnosis and effective treatment have been described to be associated with a better prognosis of FMD, ultimately reducing the burden on the National Health Services.

## Methods and materials

### Participants

Twelve consecutive patients with FMD were recruited at the tertiary level neuropsychiatric outpatient clinic of San Paolo Hospital in Milan, Italy. Diagnosis of FMD was made by a neurologist and a psychiatrist according to DSM-5 and to Gupta and Lang diagnostic criteria [18] with the presence of distractibility maneuvers and the demonstration of positive signs [19]. Twenty healthy subjects were recruited via word-of-mouth and served as control group (CTR). Their health state was investigated through a detailed anamnestic interview.

All participants signed a written informed consent. The study was conducted in accordance with the Declaration of Helsinki and approved by the Ethics Committee of “Milano Area 1” (“Registro Sperimentazioni n. 2020/ST/284” on 02/03/2022, Protocol N0010811). All procedures and methods were performed in accordance with the relevant international guidelines and regulations to reduce the physical discomfort of the participants.

### Psychometric assessment

All participants completed the Beck Anxiety Inventory (BAI) [20], the Beck Depression Inventory – II (BDI-II) [21] and two validated self-report questionnaires. The BAI measures the severity of anxiety symptoms experienced in the past week. It consists of 21 items, to be replied to on a scale from 0 (“Not at all”) to 3 (“Severely – It bothered me a lot”). A Total Score was calculated summing the answers of all the items, and participants were labeled on a severity scale, as follows: 0–7: no/minimal anxiety; 8–15: mild anxiety; 16–25: moderate anxiety; 26–63: severe anxiety. The BDI-II assesses symptoms of depression occurring in the last two weeks; it consists of 21 items, to be replied to on a scale from 0 (“Not at all”) to 3 (“Completely”). A Total Score was calculated summing the answers of all the items, and participants were labeled on a severity scale, as follows: 0–13: no/minimal depression; 14–19: mild depression; 20–29: moderate depression; 30–36: severe depression. Moreover, patients with FMD completed the Toronto Alexithymia Scale – 20 items (TAS-20), a multidimensional self-report scale of alexithymia, assessing: “Difficulties Identifying Feelings”, “Difficulties Describing Feelings” and “Externally-Oriented Thinking”; a Total Score was also calculated, and participants scoring equal or above the cut-off of 61 were labeled as alexithymic [22].

### Experimental procedure and sample collection

The experiment took place in a medical studio within San Paolo Hospital in Milan (Italy). Upon arrival, participants were given the information sheet and were asked to sign the consent form. They were invited to sit comfortably in front of a table and completed the aforementioned self-report questionnaires. Then, approximately 6 mL of venous blood was drawn from an antecubital vein, with subjects sitting. Samples were collected in lithium-heparinized and EDTA tubes, (Vacutainer, Becton Dickinson, USA). Blood was separated by centrifuge (5702 R, Eppendorf, Germany) at 3000 rpm × 10 min at 4 °C. All samples were then stored in multiple aliquots at −80 °C until assayed. Samples were thawed only once before analysis, performed within 1 month from the collection. Finally, urine was collected by voluntary voiding in a sterile container and stored in multiple aliquots at −20 °C until assayed and thawed only before analysis.

### Biomarker analysis

#### Glutamate

Glutamate concentration in plasma was measured using a glutamate assay kit (Glutamate ELISA KIT, LDS, Germany) according to the manufacturer’s instructions. Detection limits were 2.04 μM L^−1^. The amount of glutamate was quantified by colorimetric spectrophotometry at 450 nm. Measurements are expressed as μM L^−1^.

#### Brain-derived neurotrophic factor (BDNF)

BDNF was detected in plasma using ELISA method according to the manufacturer’s instructions (Human BDNF, Abcam, USA). Tertiary antibodies were conjugated to horseradish peroxidase. Wells were developed with tetramethylbenzidine and measured at 450 nm. BDNF content was quantified against a standard curve calibrated with known amounts of protein. The detection limits were 2.4 pg mL^−1^. Measurements are expressed as ng mL^−1^.

#### Dopamine

Dopamine in plasma was determined by a kit (cat no EU0392; FineTest, Wuhan, China) based on competitive-ELISA detection method. Analysis was carried out according to the manufacturer’s instructions. Dopamine concentration was determined using a standard curve. Samples and standards were read spectrophotometrically at a wavelength of 450 nm.

#### ROS

An Electron Paramagnetic Resonance (EPR) spectroscopy X-band (E-Scan – Bruker BioSpin, GmbH, MA USA), equipped with a Temperature & Gas Controller “BIO-III” (Noxigen Science Transfer & Diagnostics GmbH, Germany), was adopted to assess ROS at 37 °C. The method was previously described [23–28]. Spin probe CMH (1-hydroxy-3-methoxycarbonyl-2,2,5,5-tetramethylpyrrolidine) was used for ROS determination [29] by stable radical CP· (3-Carboxy2,2,5,5-tetramethyl-1-pyrrolidinyloxy) as external reference to convert ROS determinations in absolute quantitative values (μmol min^−1^). Spectra acquired were recorded and analyzed in duplicate using Win EPR software (version 2.11) standardly supplied by Bruker.

#### 8-Isoprostane

Lipid peroxidation was measured on urine by immunoassay of 8-isoprostane concentration (8-iso-PGF2α) (Cayman Chemical, Ann Arbor, MI, USA) as previously described [28]. Samples and standard were read at a wavelength of 512 nm.

#### Nitric oxide pathway

Nitric oxide metabolism determination (nitrite (NO_2_^−^) + nitrate (NO_3_^−^) = (Nox)) levels determination in urine was performed by the spectrophotometric method to Griess reagent [30], utilizing a commercial colorimetric assay kit (Cayman Chemical, USA) as previously reported.

Inducible nitric oxide synthase (iNOS) expression was assessed in plasma by commercially assay EIA kit (cat no EH0556; FineTest, Wuhan China). This assay was based on sandwich enzyme-linked immune-sorbent assay technology. The analysis was carried out according to the manufacturer’s instructions. NOS2/iNOS protein synthesis was determined using a standard curve. Samples and standards were read at a wavelength of 450 nm.

The concentration of 3-NT in plasma was measured by competitive-ELISA method, using an assay kit (cat no EU2560; FineTest, Wuhan, China). The analysis was carried out in accordance with the manufacturer’s instructions, and the 3-NT concentration was measured spectrophotometrically at a wavelength of 450 nm by comparing the samples’ OD (optical density) to a standard curve.

All samples’ determinations assessed by immune or enzymatic methods were carried out using a microplate reader spectrophotometer (InfiniteM200, Tecan, Austria) in duplicate, and the inter-assay coefficient of variation was in the range indicated by the kit’s manufacturer.

#### Thiols determination

Total oxidized aminothiols were measured in the erythrocytes (RBC) according to previously validated methods [31, 32] Thiols separation was performed at room temperature by isocratic HPLC analysis on a Discovery C-18 column (250 × 4.6 mm I.D, Supelco, Sigma-Aldrich, St. Louis, MOS, USA). Fluorescence intensities were measured with an excitation wavelength at 390 nm and an emission wavelength at 510 nm, using a fluorescence spectrophotometer (Jasco, Japan). A standard calibration curve was used.

#### Creatinine, neopterin, and uric acid concentration

Urinary creatinine and neopterin concentrations were measured by high-pressure liquid chromatography (HPLC) method as previously described [33]. Also, uric acid levels in urine were determined by Varian instrument (pump 240, autosampler ProStar 410) coupled to a UV-VIS detector (Shimadzu SPD 10-AV, λ = 240 nm) after centrifugation at 13,000 rpm for 5 min at 4 °C. Analytic separations were performed at 50 °C on a 5 µm Discovery C18 analytical column (250 × 4.6 mm I.D., Supelco, Sigma-Aldrich) at a flow rate of 0.9 mL min^−1^. The calibration curves were linear over the range of 0.125–1 μmol L^−1^, of 0.625–20 mmol L^−1^, and of 1.25–10 mmol L^−1^ for neopterin, uric acid and creatinine levels, respectively. Inter-assay and intra-assay coefficients of variation were <5%.

### Statistical analysis

Statistical analyses were performed with SPSS 27 (Statistical Package for Social Science). Significance level was set at α (0.05), and all tests were two-tailed.

First, Kolmogorov–Smirnov test was implemented to assess whether each variable followed a normal distribution. Second, descriptive statistics were calculated, and a series of *t* test for independent samples was run to test the differences between FMD patients and CTR for demographic and psychometric variables; *t* test results are reported according to Levene’s test for homogeneity of variances. With respect to biological variables, a series of ANCOVA were run with Groups (CTR vs FMD) as between-subject factor, the biological variables as dependent variables (separately), and the Total Scores of the BAI and the BDI-II to control for levels of anxiety and depression, respectively. Finally, within the FMD group, we investigated possible associations between the biomarkers shown to be significantly different between patients with FMD and CTR and the psychometric assessment; three multiple linear regressions were run with: (i) glutamate, BDNF, and Dopamine (separately) as dependent variable; (ii) the BDI-II and BAI Total Scores and the TAS-20 subscales (DIF, DDF, EOT) as independent variables.

## Results

### Sociodemographic and clinical information

Groups were balanced for age (*t* = −1.364, df = 30, *p* = 0.183, 95% [−22.35; 4.45], gender (*χ* (1) = 0.711, *p* = 0.399) and BMI (*t* = −0.806, df = 30, *p* = 0.427, 95% CI [−4.5; 1.9] Further details regarding anthropometric and physiological parameters in FMD patients and CTR group are reported in Table 1.

**Table 1:** Demographic information.

	CTR	FMD	*t*	df	*p*	95% CI
Sex (M/F)	4/16	4/8	0.711	1	0.399	NA
Age (years)	46.30 ± 16.99	55.25 ± 19.53	−1.364	30	0.183	[−22.35; 4.45]
Weight (kg)	66.53 ± 12.82	68.68 ± 13.49	−0.450	30	0.656	[−11.89; 7.6]
Height (cm)	168 ± 7.71	166.3 ± 7.48	0.628	30	0.535	[−3.94; 7.44]
BMI (kg m^−2^)	23.56 ± 4.17	24.84 ± 4.65	−0.806	30	0.427	[−4.53; 1.96]
SaO_2_ (%)	98.2 ± 0.83	98.33 ± 0.89	−0.428	30	0.672	[−0.77; 0.5]

*CI* confidence interval, *CTR* healthy controls, *df* degrees of freedom, *F* female, *FMD* functional movement disorders, *M* male; *p* *p* value, *SaO*_*2*_ oxygen saturation, *t* Student’s *t*.

### Psychometric assessment

Patients with FMD scored significantly higher than CTR at the BAI Total Score (*t* = −4.573, df = 13.5, *p* = 0.002, 95% CI = [−10.039; −5.594], Cohen’s *D* = 1.67, Hedges’ *G* = 1.628) and at the BDI-II Total Score (*t* = −2.55, df = 30, *p* = 0.016, 95% CI [−13.17; −1.46], Cohen’s *D* = 0.932, Hedges’ *G* = 0.908). Five patients with FMD (41.7%) scored above the cut-off at the TAS-20 Total Score. Further details are reported in Table 2.

**Table 2:** Psychometric assessment.

	CTR	FMD	*t*	df	*p*	95% CI
BDI-II Total Score, mean ± SD	6.10 ± 6.38	13.42 ± 9.90	−2.55	30	0.016	[−13.17; −1.46]
BAI Total Score, mean ± SD	5.6 ± 4.76	18.42 ± 11.02	−3.819	13.5	0.002	[−20.04; −5.59]
TAS-20—Total Score, mean ± SD	NA	52.33 ± 14.5	NA	NA	NA	NA
TAS-20—alexithymic/not alexithymic	NA	5/7	NA	NA	NA	NA
TAS-20 DIF, mean ± SD	NA	19.42 ± 9.84	NA	NA	NA	NA
TAS-20 DDF, mean ± SD	NA	13.75 ± 4.14	NA	NA	NA	NA
TAS-20 EOT, mean ± SD	NA	19.17 ± 5.83	NA	NA	NA	NA

*BAI* Beck Anxiety Inventory, *BDI-II* Beck Depression Inventory, 2nd version, *CI* confidence interval, *CTR* healthy controls, *df* degrees of freedom, *FMD* functional movement disorders, *p* *p* value, *SD* standard deviation, *TAS-20* Toronto Alexithymia Scale—20 items, *TAS-20 DDF* Difficult Describing Feelings, *TAS-20 DIF* Difficult Identifying Feelings, *t* Student’s *t*.

### Levels of circulating glutamate, BDNF, and dopamine levels

Controlling for anxiety (BAI) and depression (BDI-II), levels of glutamate (*F* (1, 28) = 20.178, *p* < 0.001, *η*^2^_p_ = 0.419), BDNF (*F* (1, 28) = 9.784, *p* < 0.004, *η*^2^_p_ = 0.259) and dopamine (*F* (1, 28) = 19.134, *p* < 0.001, *η*^2^_p_ = 0.406) were significantly lower in patients with FMD than in CTR. Further details are reported in Table 3.

**Table 3:** Biomarkers.

	CTR	FMD	*F*	*p*	η2p	BAI effect (covariate)	BDI-II effect (covariate)
GLU (μM L^−1^)	77.08 ± 21.19	42.89 ± 20.97	20.178	**<0.001**	0.419	*F* = 1.313 *p* = 0.262	*F* = 0.073 *p* = 0.789
BDNF (ng mL^−1^)	1.49 ± 0.7	0.64 ± 0.4	9.784	**0.004**	0.259	*F* = 0.623 *p* = 0.437	*F* = 0.818 *p* = 0.373
DOPA (pg mL^−1^)	26.31 ± 4.8	16.80 ± 3.23	19.134	**<0.001**	0.406	*F* = 0.012 *p* = 0.912	*F* = 0.339 *p* = 0.565
ROS (μmol min^−1^)	0.174 ± 0.01	0.179 ± 0.01	0.09	0.766	0.003	*F* = 3.86 *p* = 0.059	*F* = 3.227 *p* = 0.083
8-iso PGF2α (pg mg^−1^creatinine)	301.2 ± 91.67	402.3 ± 165.31	1.272	0.269	0.045	*F* = 4.449 *p* = 0.044	*F* = 5.109 *p* = 0.032
NOx (μM)	406.56 ± 210.05	406.34 ± 123.23	0.104	0.749	0.004	*F* = 0.028 *p* = 0.868	*F* = 1.045 *p* = 0.316
NO_2_ (μM)	0.63 ± 0.45	0.61 ± 0.37	0.234	0.632	0.008	*F* = 0.68 *p* = 0.417	*F* = 0.324 *p* = 0.573
iNOS (I.U. mL^−1^)	35.77 ± 6.42	37.996 ± 12.14	1.362	0.253	0.046	*F* = 2.284 *p* = 0.142	*F* = 1.652 *p* = 0.209
3-NT (ng mL^−1^)	52.04 ± 18.52	55.8 ± 26.24	0.11	0.743	0.004	*F* =0.017 *p* = 0.896	*F* = 0.08 *p* = 0.779
Neopterin μm mol^−1^creatinine	161.31 ± 38.5	165.76 ± 31,74	1.803	0.191	0.063	*F* = 5.055 *p* = 0.033	*F* = 2.65 *p* = 0.115
Uric acid (mM)	3.14 ± 2.05	3.03 ± 1.96	0.398	0.534	0.015	*F* = 2.718 *p* = 0.111	*F* = 1.554 *p* = 0.224
GSH (μmol L^−1^)	1467.14 ± 304.09	1600.64 ± 632.51	0.202	0.657	0.007	*F* = 1.794 *p* = 0.060	*F* = 3.916 *p* = 0.058
Hcy (μmol L^−1^)	2.79 ± 0.62	3.21 ± 0.6	0.696	0.411	0.024	*F* = 0.359 *p* = 0.554	*F* = 0.051 *p* = 0.822
Cys (μmol L^−1^)	64.56 ± 12.12	68.31 ± 10.19	1.212	0.28	0.041	*F* = 0.128 *p* = 0.724	*F* = 2.362 *p* = 0.136
CysGly (μmol L^−1^)	2.66 ± 0.5	2.78 ± 0.83	0.052	0.821	0.002	*F* = 1.133 *p* = 0.0.296	*F* = 2.47 *p* = 0.127

All statistics are reported with degrees of freedom = (1, 28).

*3-NT* 3-nitrotyrosine, *8-iso PGF2α* 8-isoprostane, *BDNF* brain-derived neurotrophic factor, *CTR* healthy controls, *Cys* cysteine, *CysGly* cysteinylglycine, *DOPA* dopamine, *FMD* functional movement disorders, *GLU* glutamate, *GSH* glutathione, *Hcy* homocysteine, *iNOS* inducible nitric oxide synthase, *NO*_*2*_ nitrite, *NOx* nitric oxide metabolites [(NO_2_^−^) nitrite + nitrate (NO_3_^−^)], *p* *p* value, *ROS* reactive oxygen species, *SD* standard deviation, *η*^*2*^_*p*_ partial eta squared.

Bold values indicates statistically significant *p* values (*p* < 0.05).

### Assessment of oxidative stress and redox status

Controlling for anxiety (BAI) and depression (BDI-II), no significant differences (all *p* > 0.05) in the levels of lipid peroxidation, neopterin, creatinine and uric acid between FMD and CTR groups were recorded. Furthermore, no significant differences (all *p* > 0.05) between the two groups emerged with respect to total thiols (cysteine (Cys); cysteinylglycine (CysGly); homocysteine (Hcy); glutathione (GSH) concentrations in red blood cells). Further details are reported in Table 3.

### Availability of nitric oxide

Controlling for anxiety (BAI) and depression (BDI-II), concentrations of iNOS, Nox, NO_2_ and 3-NT in patients with FMD were not significantly different (all *p* > 0.05) from those in CTR. Further details are reported in Table 3.

### Regression analysis within the FMD sample

#### Glutamate

The TAS-20 subscale “Difficult Describing Feelings” was positively associated with the levels of Glutamate (*b* = 4.407, *t* = 3.432, *p* = 0.014). The other TAS-20 subscales, as well as the levels of anxiety and d (as per BAI and BDI-II) were not associated with Glutamate levels.

#### BDNF

None of the psychometric variables were associated with BDNF levels.

#### Dopamine

Dopamine levels were positively associated with: TAS-20 “Difficult Identifying Feelings” (*b* = 0.284, *t* = 4.201, *p* = 0.006), TAS-20 “Difficult Describing Feelings” (*b* = 0.339, *t* = 2.58, *p* = 0.042); they were negatively associated with: BAI (*b* = −0.425, *t* = −4.663, *p* = 0.003), TAS-20 “Externally Oriented Thinking” (*b* = −0.589, *t* = −5.309, *p* = 0.002); they were not associated with BDI-II.

See Table 4 for further details.

**Table 4:** Regression analysis within the FMD sample.

		Unstandardized coefficient		Standardized coefficient: beta	*t*	*p*	95%CI		Correlations		
		*B*	Standard error				Inf. lim.	Sup. lim.	Zero-order	Partial	Part
GLU	Intercept	−5.818	21.864		−0.266	0.799	−59.317	47.681			
	BDI Total Score	1.772	0.847	0.836	2.090	0.082	−0.302	3.845	0.373	0.649	0.441
	BAI Total Score	−0.782	0.890	−0.411	−0.878	0.414	−2.959	1.396	0.425	−0.338	−0.185
	TAS-20 DIF	−0.015	0.661	−0.007	−0.022	0.983	−1.632	1.603	0.116	−0.009	−0.005
	TAS-20 DDF	4.407	1.284	0.869	3.432	**0.014**	1.265	7.549	0.665	0.814	0.725
	TAS-20 EOT	−1.094	1.085	−0.304	−1.009	0.352	−3.749	1.560	−0.060	−0.381	−0.213
BDNF	Intercept	0.266	0.694		0.383	0.715	−1.432	1.963			
	BDI Total Score	−0.036	0.027	−0.901	−1.354	0.225	−0.102	0.029	−0.177	−0.484	−0.476
	BAI Total Score	0.034	0.028	0.945	1.214	0.270	−0.035	0.103	0.086	0.444	0.427
	TAS-20 DIF	−0.011	0.021	−0.277	−0.536	0.611	−0.063	0.040	0.088	−0.214	−0.189
	TAS-20 DDF	−0.018	0.041	−0.184	−0.438	0.677	−0.117	0.082	0.115	−0.176	−0.154
	TAS-20 EOT	0.036	0.034	0.530	1.057	0.331	−0.048	0.121	0.122	0.396	0.371
DOPA	Intercept	23.383	2.238		10.449	0.000	17.908	28.859			
	BDI Total Score	0.176	0.087	0.538	2.027	0.089	−0.036	0.388	−0.501	0.638	0.284
	BAI Total Score	−0.425	0.091	−1.448	−4.663	**0.003**	−0.648	−0.202	−0.429	−0.885	−0.654
	TAS-20 DIF	0.284	0.068	0.865	4.201	**0.006**	0.119	0.450	−0.054	0.864	0.589
	TAS-20 DDF	0.339	0.131	0.434	2.580	**0.042**	0.018	0.661	0.155	0.725	0.362
	TAS-20 EOT	−0.589	0.111	−1.063	−5.309	**0.002**	−0.861	−0.318	−0.480	−0.908	−0.744

*BAI* Beck Anxiety Inventory, *BDI-II* Beck Depression Inventory, 2nd version, *BDNF* brain-derived neurotrophic factor, *CI* confidence interval, *DOPA* dopamine, *GLU* glutamate, *FMD* functional movement disorders, *p* *p* value, *TAS-20* Toronto Alexithymia Scale—20 items, *TAS-20 DDF* Difficult Describing Feelings, *TAS-20 DIF* Difficult Identifying Feelings.

Bold values indicates statistically significant *p* values (*p* < 0.05).

## Discussion

The main result of this study, which is a first step in the direction of establishing possible biomarkers for FMD, is that levels of glutamate, BDNF and dopamine were significantly lower in the blood of patients with FMD than in healthy controls. To the best of our knowledge, this is the first study assessing circulating biological markers in a specific population of patients with FND-motor subtypes. Previous studies have assessed these biomarkers in other psychiatric conditions, overall arguing in favor of their implications in the pathogenesis of several neuropsychiatric conditions, such as depression, anxiety, and schizophrenia. Our results lead us to hypothesize, first, that a dysregulation of the glutamatergic pathway would be present in patients with FMD, possibly representing a biomarker of disease. These findings are in line with our previous studies arguing in favor of a glutamatergic dysfunction in FMD: compared to CTR, FMD patients showed increased levels of Glx in the anterior cingulate cortex/medial prefrontal cortex (ACC/mPFC) and lower cerebrospinal fluid CSF levels of glutamate [7, 8]. Our results also fit with a recent hypothesis proposed by Komagamine et al. [34], according to which, based on Charcot’s observations and current progress in molecular biology (e.g., the identification of glutamate/NMDA receptor system dysfunction in drug withdrawal syndrome), patients with conversion disorders and patients under hypnosis can also have hypofunction of the glutamatergic system. Moreover, we found a positive association between the TAS-20 subscale “Difficult Describing Feelings” and levels of glutamate in FMD patients, which is a result again in line with the positive correlation found by our previous study between high scores of alexithymia and anxiety and altered glutamate concentrations in the limbic system of patients with FMD [7]. These results add some evidence to the extensive recently developing literature that involves glutamate system in the pathogenesis of anxiety and its related disorders. Limbic and paralimbic brain regions (amygdala, hippocampus, anterior cingulate cortex, medial prefrontal cortex, insula) are known as the anatomical core of fear/anxiety and are richly innervated by glutamatergic pyramidal cells. At these levels, glutamate acts through direct activation of its ionotropic (NMDA and AMPA/kainate) and metabotropic receptors, and by modulating the release of other neurotransmitters involved in stress-response like serotonin, dopamine, monoamine, and GABA. Neuroimaging studies have shown structural or functional alterations in glutamate-rich regions like the amygdala, hippocampus, and ACC in patients with anxiety disorders supporting the role of glutamatergic hyperactivation in causing anxiety [35]. Although here we will not be able to establish a direct causative effect between possible glutamatergic dysfunction and FMD, we might propose the role of glutamatergic dysfunction as a potential biomarker of the disease. Moreover, our findings might prompt research to test novel pharmacological approaches with drugs acting on N-methyl-D-aspartate receptors (ketamine, memantine, D-cycloserine) in FMD.

With respect to BDNF, we found a decreased blood level in patients with FMD compared to healthy controls. This is in line with the study by Deveci et al. [8], which found a decreased level of BDNF in patients with FND, compared to CTR. They also found that serum BDNF levels were not significantly different in patients with FND and major depressive disorder, arguing in favor of a transdiagnostic role of BDNF in terms of representing a possible biological marker for stress-related psychiatric disorders. This hypothesis is also in line with the study by LaFrance et al. [36], where patients with psychogenic nonepileptic seizures were found to have lower serum glutamate levels than healthy controls, but similar glutamate levels to patients with epilepsy, suggesting again the possible role of BDNF decrease in different neuropsychiatric conditions. In this view BDNF should not be considered a specific biomarker for FND, but a more general diagnostic tool detectable in different neuropsychiatric conditions sharing stress-related pathogenesis.

Finally, our results showed a lower level of dopamine in patients with FMD than in healthy controls. It is well known that dopamine is implicated in the pathogenesis of different movement disorders, such as Parkinson’s disease. Moreover, dopamine seems to have a key role also in anhedonia, which is a symptom both of depression and Parkinson’s disease: Belujon and Grace [12] described how downregulation of the dopaminergic system can constitute a mechanism underlying anhedonia (inability to feel satisfaction, contentment, or interest in usual pleasant activities [35]). Glutamatergic and dopaminergic systems are closely interconnected, and this might have relevance also in terms of treatment options for FMD: in patients suffering from depression, ketamine, a non-competitive, glutamatergic NMDA-receptor antagonist, has been described to have rapid and prolonged antidepressant effects after a single dose [37, 38]. It is now well-established that ketamine increases AMPA-dependent glutamate transmission, particularly in the prefrontal cortex, either via an increased synthesis of synaptic proteins in pyramidal neurons or via antagonism of NMDA-receptor on cortical interneurons [39]. The cellular and molecular effects of ketamine have been extensively studied in the past years [40]. At the circuit level, Belujon and Grace [41] showed that ketamine injection restored the activity of ventral tegmental area dopaminergic neurons and restored synaptic dysfunction observed in stress-induced depression in rats previously exposed to learn helplessness behavior, to a level comparable with control and non-helpless animals. Understanding some of ketamine antidepressant properties at a system level will help in finding new treatment strategies that could be effective also in patients with FMD.

Our study has the following limitations; first, the small sample size that might influence the generalizability of our results; second, our sample encompasses only patients with FND-motor subtypes, excluding patients with PNES and sensory FND; third, we only detected biological markers in the blood/urine and not in the CSF or in the brain of patients and controls; fourth, we did not include a group of patients with organic neurological diseases as a control group. Finally, we did not consider the presence of other psychiatric and organic comorbidities (except for anxiety and depression levels), nor the potential effect of the medication that these patients were treated with, given their heterogeneity and the small sample size. This might have influenced our results, partially driving both the differences between groups and the associations that we appreciated in the FMD groups, instead of representing intrinsic FMD mechanisms. Hence, our finding should be considered as an initial step in the attempt to establish biomarkers for FMD, which should be confirmed by future larger and in-depth studies.

## Conclusions

In conclusion our study revealed a significant decreased level of glutamate, BDNF and dopamine in the blood of patients with FMD when compared to healthy controls. Deepening the knowledge of FMD, in terms of finding disease biomarkers and subsequently drawing specific treatments, might not only help defining potential mechanisms underlying the disorder, but also have clinical implications, both from a diagnostic and therapeutic angle.

## Data Availability

Anonymized data will be shared upon request from any qualified investigator.
